# Measuring hand hygiene opportunities per hour across two neonatal intensive care units

**DOI:** 10.1017/ash.2023.319

**Published:** 2023-09-29

**Authors:** Eugene Lee, Souad Al-Muthree, Paige Reason, Meghan Donohue, Michael Dunn, Meghan Statchuk, Sarah Khan, Shikha Gupta-Bhatnagar, Salhab El-Helou, Jerome Leis, Dominik Mertz

## Abstract

**Background:** To estimate hand hygiene compliance using electronic hand hygiene monitoring, the number of hand hygiene opportunities (HHOs) per period must be known in a given setting. Data on the number of HHOs in a neonatal ICU (NICU) are limited. We measured HHOs per hour and identified factors that may influence the number of HHOs per hour to calibrate compliance estimates for electronic hand hygiene monitoring. **Methods:** The study was conducted in 2 large NICUs in Ontario, Canada (72 and 42 beds, respectively). We centrally trained observers to identify HHOs using the Ontario-based “Four Moments of Hand Hygiene,” which is similar to combining moments 4 and 5 of the WHO “Five Moments of Hand Hygiene.” To apply the moments of hand hygiene to the NICU setting, the following modifications were made: moment 1 was entering the incubator or contact with anything within the ‘baby space’ directly around the incubator, and moment 4 was when hands exited the incubator and, as such, the ‘baby space.’ Using a standardized tool, the investigators conducted direct observation of HHOs during randomized observation periods from July 1, 2022, to January 9, 2023. In addition to HHOs, data on covariables potentially associated with the frequency of HHOs were collected: time and day of the week, acuity, additional precautions, corrected gestational age, and private versus multibed room or open pod. **Results:** We audited HHOs for 146 hours including 26 at site A and 120 at site B. Overall, 804 HHOs (69.2%) occurred during weekdays and 739 (63.6%) occurred during day shifts from 7:00 a.m. to7:00 p.m. The most frequent moments of hand hygiene were moment 1 (47.8%, before contact) and moment 4 (36.8%, after contact). The average numbers of HHOs were 7.8 per hour overall, 7.6 per hour on weekdays, 7.7 per hour on weekends, 8.8 per hour on day shifts, and 6.8 per hour on night shifts. The breakdown of HHOs by profession was 92.8% nurses, 0.6% physicians, 4.5% allied health, and 2.1% for others. **Discussion:** The rate of HHOs in NICU varied over a 24-hour period and was similar between 2 different NICUs. Evenings and weekends had considerably fewer average HHOs, and peaks were observed following nursing shift changes. The rate of HHOs may be influenced by other factors including unit design, patient acuity, and use of transmission-based precautions. Further analysis using a Poisson regression model will help to explore these factors and to calibrate electronic monitoring for this population.

**Figure:**
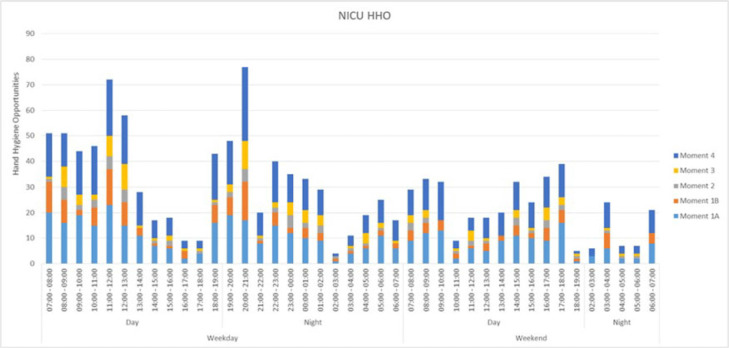

**Disclosures:** None

